# Community Structure, Dynamics and Interactions of Bacteria, Archaea and Fungi in Subtropical Coastal Wetland Sediments

**DOI:** 10.1038/s41598-018-32529-5

**Published:** 2018-09-26

**Authors:** Man Kit Cheung, Chong Kim Wong, Ka Hou Chu, Hoi Shan Kwan

**Affiliations:** School of Life Sciences, The Chinese University of Hong Kong, Hong Kong SAR, China

## Abstract

Bacteria, archaea and fungi play crucial roles in wetland biogeochemical processes. However, little is known about their community structure, dynamics and interactions in subtropical coastal wetlands. Here, we examined communities of the three kingdoms in mangrove and mudflat sediments of a subtropical coastal wetland using Ion Torrent amplicon sequencing and co-occurrence network analysis. Bacterial, archaeal and fungal communities comprised mainly of members from the phyla Proteobacteria and Bacteroidetes, Bathyarchaeota and Euryarchaeota, and Ascomycota, respectively. Species richness and Shannon diversity were highest in bacteria, followed by archaea and were lowest in fungi. Distinct spatiotemporal patterns were observed, with bacterial and fungal communities varying, to different extent, between wet and dry seasons and between mangrove and mudflat, and archaeal community remaining relatively stable between seasons and regions. Redundancy analysis revealed temperature as the major driver of the seasonal patterns of bacterial and fungal communities but also highlighted the importance of interkingdom biotic factors in shaping the community structure of all three kingdoms. Potential ecological interactions and putative keystone taxa were identified based on co-occurrence network analysis. These findings facilitate current understanding of the microbial ecology of subtropical coastal wetlands and provide a basis for better modelling of ecological processes in this important ecosystem.

## Introduction

Bacteria, archaea and fungi in wetland sediments play crucial roles in biogeochemical processes including carbon, nitrogen and sulphur cycling. Information on their community structure and dynamics are therefore crucial to understanding the microbial ecology of wetlands and to improve global modelling of climate change^1^. In the past decade, the advent of high-throughput next-generation sequencing (NGS) technologies has greatly facilitated our understanding of microbial communities in various environments. However, the majority of NGS studies in wetlands to date have been conducted in temperate marshes and/or focused on the bacterial kingdom only^2–4^. Information on the less abundant yet comparably important archaeal and fungal communities in subtropical mangrove wetlands remain scarce^5–8^, albeit the occurrence of a global marsh-to-mangrove transition as a result of climate change^9^. Without more in-depth knowledge on these two often-overlooked kingdoms, our understanding of wetland microbial ecology will remain biased and incomplete.

Apart from knowing the structure and dynamics of microbial communities, a more integrated understanding of microbial communities and the ecological rules guiding community assembly could be achieved using network analysis^10^. Co-occurrence networks can unveil ecologically meaningful interactions and help ascertain the functional roles or environmental niches occupied by uncultured microorganisms^10^. Until now, co-occurrence networks for wetlands have only been constructed for bacteria and fungi in salt marsh soils^4,7^. Ecological relationships among uncultured microorganisms residing in subtropical mangrove wetland sediments remain largely elusive.

In this study, we examined in detail for the first time the bacterial, archaeal and fungal communities in subtropical coastal wetland sediments using NGS and network analysis. Our major aim was to provide a comprehensive understanding of the community structure, dynamics and interactions of major microbial players from all three kingdoms in this important ecosystem. As rapid sea-level rise is expected to accelerate the conversion of coastal mangrove and marshes to bare mudflat or open water^11^, we were in particular interested to compare the microbial communities in mangrove and mudflat sediments. Specific objectives were: (1) to elucidate the diversity and composition of bacterial, archaeal and fungal communities residing in sediments of Mai Po wetland in subtropical Hong Kong using Ion Torrent amplicon sequencing; (2) to examine the spatial (mangrove vs mudflat) and temporal (wet vs dry season) variations of the microbial communities; (3) to reveal biotic and abiotic factors that shape the microbial communities; and (4) to infer potential ecological interactions and putative keystone taxa using co-occurrence network analysis.

Distance from the shore is associated with the impact of tides on the affected sediments whereas the presence/absence of vegetation could affect the organic carbon content and oxygen availability of the underneath sediments^12^. Moreover, seasonality affects the temperature of surface water and sediment, the amount of rainfall, and thus water salinity, as well as the phenology of vegetation, including the amount of leaf litter-fall, which in turn affects the amount of organic carbon input to the underneath sediment^13^. Therefore, we hypothesized that the structure of microbial communities differs between the seaward bare mudflat and the landward mangrove-vegetated sediments, as well as between the hot wet and cool dry seasons. We also anticipated that the microbial communities are predominantly driven by abiotic factors but that interkingdom biotic factors also explain variation^14^.

## Results

### Physicochemical parameters

Sediment samples collected in this study were high in sulphur content (mean TS = 0.77%) (Supplementary Table S1). The mangrove samples were slightly acidic whereas the mudflat samples were slightly alkaline. Negative redox potential (ORP) values and a strong odour of H_2_S suggested that the mudflat samples were anoxic. In both seasons, the mangrove samples contained higher amount of total carbon (TC) and total nitrogen (TN) than the mudflat samples (t-test, *P* < 0.05). By contrast, the mudflat samples were more reducing and richer in total sulphur (TS) compared to the mangrove samples in both seasons (t-test, *P* < 0.05). Comparison between seasons revealed significant difference only in ORP and TN of mudflat samples (t-test, *P* < 0.001).

### Microbial community composition

**Figure 1:**
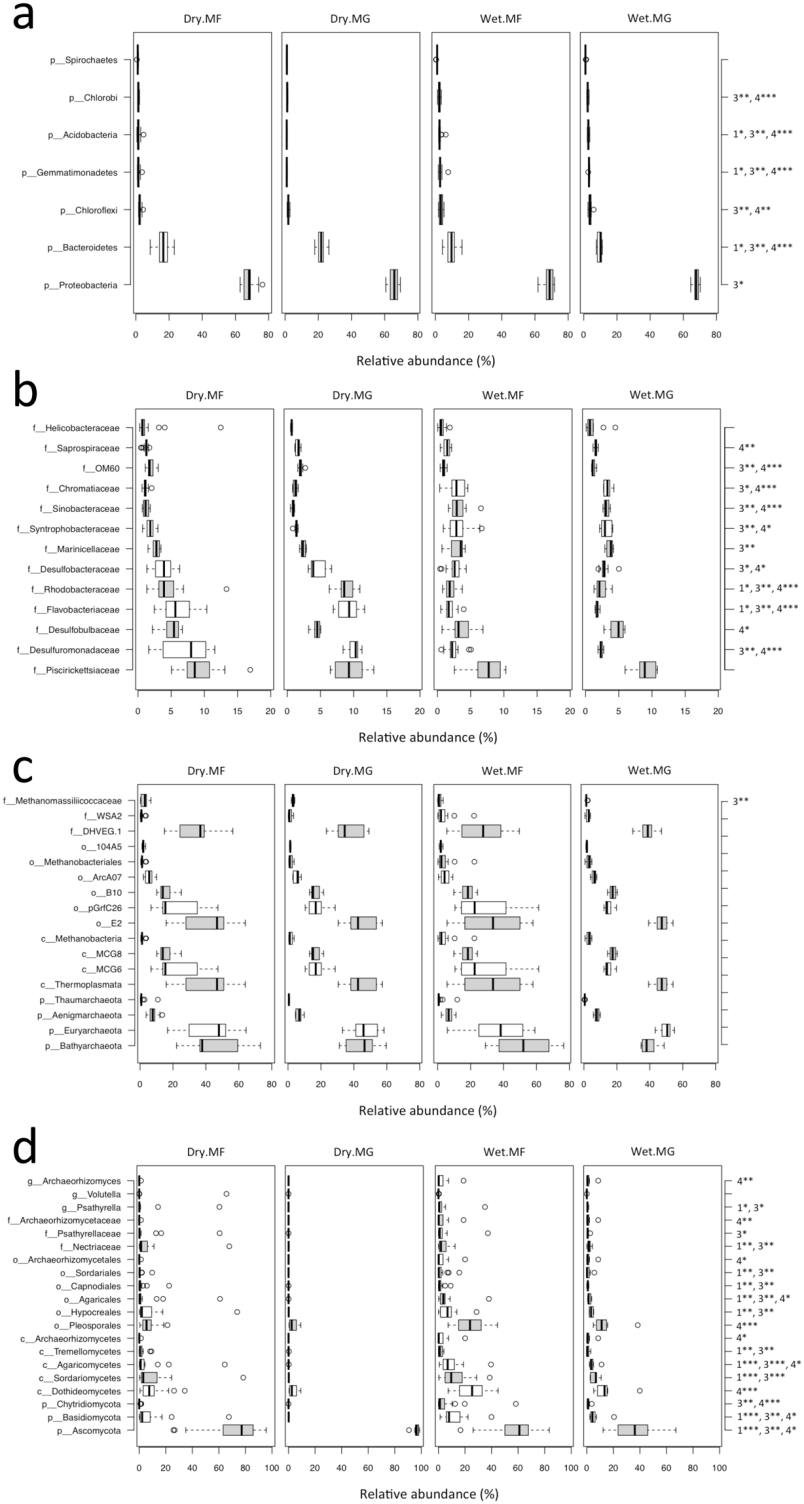
Relative abundance of dominant bacterial (**a**) phyla, (**b**) families, and multiple taxonomic ranks of (**c**) archaea and (**d**) fungi. Only groups with an average relative abundance ≥1% are shown here. Taxonomic ranks are designated by p = phylum, c = class, o = order, f = family, and g = genus. Remarks to the right of the box plots represent statistical significance between sample groups (1: Dry.MF vs Dry.MG, 2: Wet.MF vs Wet.MG, 3: Dry.MG vs Wet.MG, 4: Dry.MF vs Wet.MF; *P < 0.05, **P < 0.01, ***P < 0.001). MF: mudflat, MG: mangrove.

The bacterial community was dominated by the phyla Proteobacteria and Bacteroidetes, comprising about 68% and 14%, respectively (Fig. 1a, Supplementary Fig. S1a). Piscirickettsiaceae (9%), Desulfuromonadaceae (5%), Desulfobulbaceae (4%), Flavobacteriaceae (4%), Rhodobacteraceae (4%), and Desulfobacteraceae (3%) were the most dominant families and tended to be more represented in the dry season (*P* < 0.05) (Fig. 1b). By contrast, the less-represented families Marinicellaceae (3%), Syntrophobacteraceae (2%), Sinobacteraceae (2%), and Chromatiaceae (2%) were more represented in the wet season (*P* < 0.05).

The archaeal community was dominated by the phyla Bathyarchaeota and Euryarchaeota, comprising about 47% and 43%, respectively (Fig. 1c, Supplementary Fig. S1b). None of the dominant groups at different taxonomic levels showed significant difference in relative abundance between seasons or regions (*P* > 0.05), with family Methanomassiliicoccaceae (2%) being the sole exception (Dry.MG vs Wet.MG, *P* < 0.01) (Fig. 1c).

The fungal community was mainly composed of the phyla Ascomycota (66%) and Basidiomycota (8%), which were more represented in the dry and wet season, respectively (*P* < 0.05) (Fig. 1d, Supplementary Fig. S1c). These two phyla also showed significant differences in relative abundance between sampling regions in the dry season (*P* < 0.001). About 23% of the total internal transcribed spacer (ITS) reads, and up to 55% of those for mangrove samples from the wet season, could not be assigned to a known fungal phylum (Supplementary Fig S1c). Most of the dominant groups at different taxonomic levels were more represented in one or both sampling regions in the wet season (*P* < 0.05) and in the mudflat compared to the mangrove region in the dry season (*P* < 0.05) (Fig. 1d).

### Alpha diversity

An average of 6,308 to 7,886 observed bacterial operational taxonomic units (OTUs) were recovered per sample after normalization to 20,310 reads (Supplementary Table S2). Average values of Shannon and phylogenetic diversity (PD) indices ranged from 10.84 to 11.58 and 361 to 429, respectively. For both mudflat and mangrove, bacterial richness and diversity in samples from the wet season were higher than those from the dry season (*P* < 0.05) (Supplementary Fig. S2a–c). Bacterial richness and diversity were also higher in mudflat than mangrove samples from the dry season (*P* < 0.05). The average number of observed OTUs of archaea per sample ranged from 1,507 to 1,713 after normalization to 20,300 reads (Supplementary Table S2). Average values of Shannon and PD indices were 7.51 to 7.93 and 94 to 102, respectively. No significant differences in archaeal richness and diversity were observed among all sample groups, with the comparison of Shannon diversity between mudflat sediments from the dry season and mangrove sediments from the wet season being the sole exception (Supplementary Fig. S2d–f). There were an average of 385 to 603 observed fungal OTUs per sample after normalization to 20,250 reads (Supplementary Table S2). Values of Shannon diversity averaged from 2.52 to 5.38. Higher fungal richness and diversity were observed in mudflat than mangrove samples from the dry season (*P* < 0.05) (Supplementary Fig. S2g,h).

### Beta diversity

**Figure 2:**
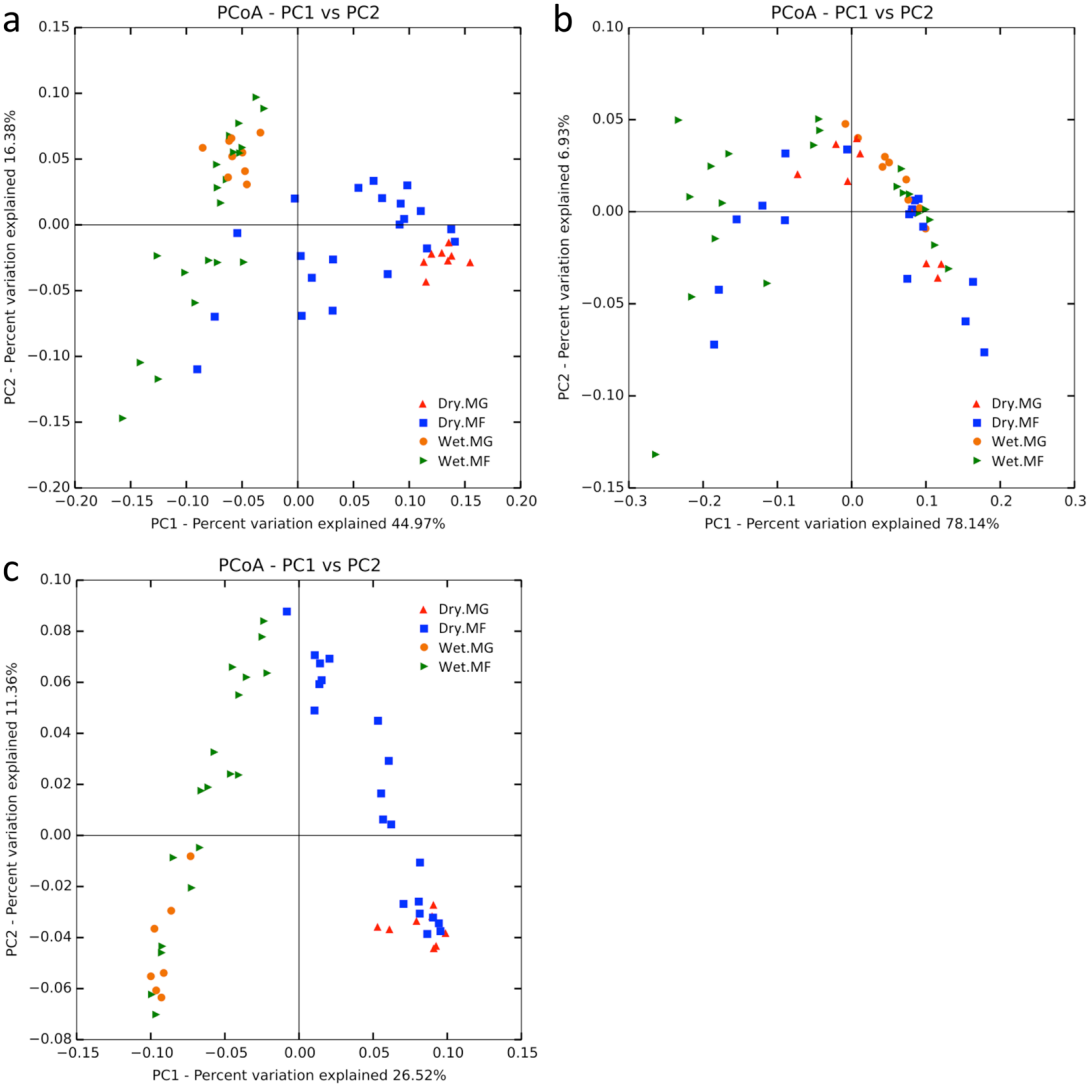
Principal coordinates analysis (PCoA) plots of (**a**) bacterial, (**b**) archaeal and (**c**) fungal communities. Weighted UniFrac distances were used for bacteria and archaea whereas Bray-Curtis distances were used for fungi. MF: mudflat, MG: mangrove.

**Figure 3:**
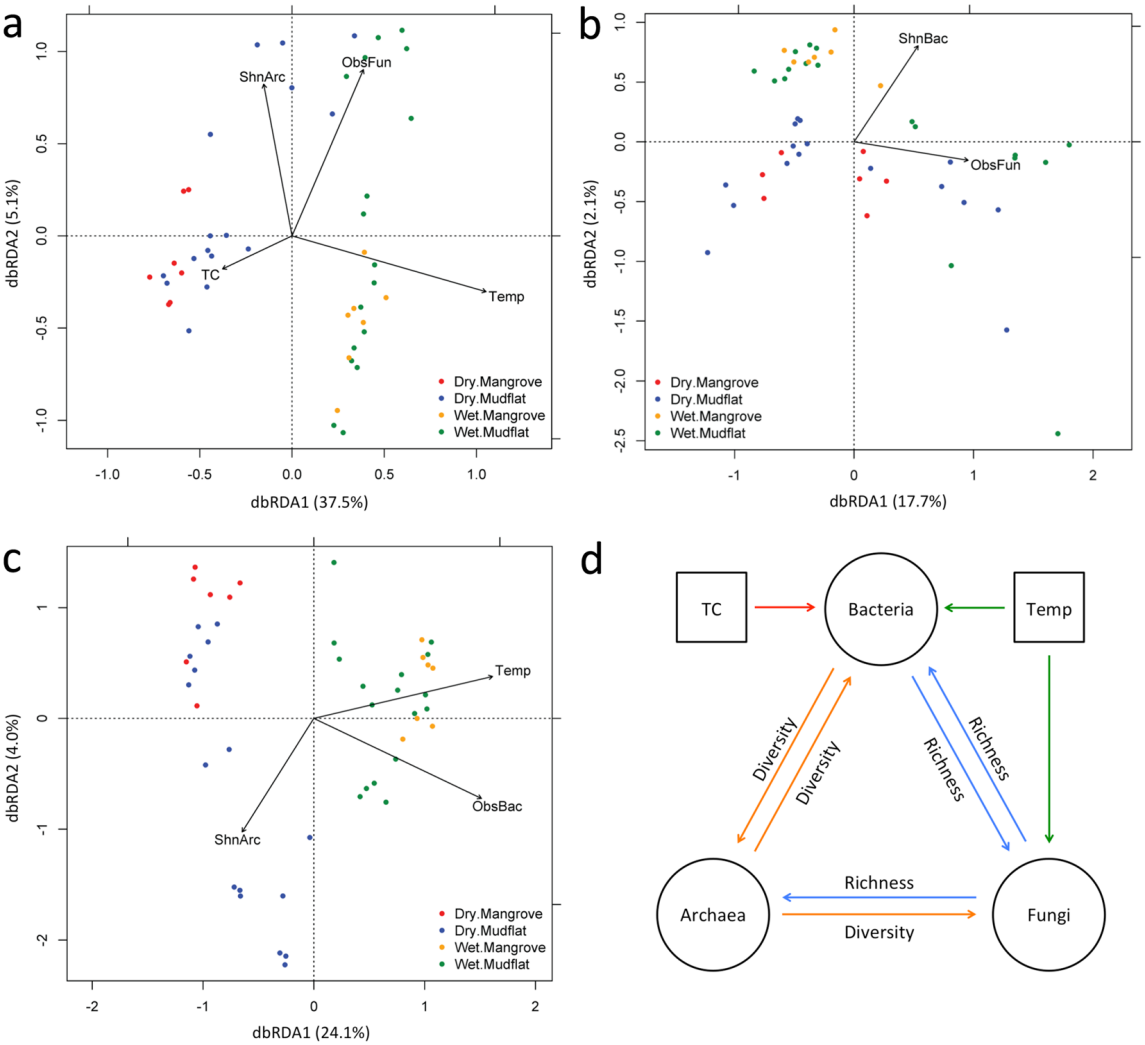
Distance-based redundancy analysis (db-RDA) biplots of (**a**) bacterial, (**b**) archaeal and (**c**) fungal communities, and (**d**) a conceptual model of factors that shape the sediment microbial communities. Percentage values on the biplot axes represent the amount of variation explained. TC: total carbon, Obs: observed number of OTUs, Shn: Shannon diversity, Bac: bacteria, Arc: archaea, Fun: fungi.

Both principal coordinates analysis (PCoA) and non-metric multidimensional scaling (NMDS) revealed similar patterns (Fig. 2, Supplementary Fig. S3), indicating that the arches in the PCoA plots were not artifacts of dimensionality reduction but a real phenomenon here. As a result, further analyses and discussions were based on the PCoA results only. Unconstrained PCoA of bacterial community revealed significant grouping of samples by season and the season × region interaction term (Fig. 2a, Table 1). However, despite a significant *P*-value (0.045) of permutational multivariate analysis of variance (PERMANOVA), the small Pseudo-*F* value (2.456) and analysis of similarities (ANOSIM) statistics (R = −0.001) obtained suggested only a weak effect of sampling region on the bacterial community. Constrained distance-based redundancy analysis (db-RDA) resulted in a model that could explain 45.3% of the total variation of the bacterial community (Table 2, Fig. 3a). Temperature was the most important factor, which alone explained 30.9% of the variation. Other significant factors that shape the bacterial community included archaeal diversity (5.5%), TC (5.3%) and fungal richness (3.5%). Based on the db-RDA results, seasonal difference of the bacterial community in both mangrove and mudflat sediments could be explained by temperature, whereas that in mangrove sediments could additionally be explained by TC and archaeal diversity (Fig. 3a).

**Table 1:** Seasonal and spatial effects on microbial communities.

Kingdom	Effect	PERMANOVA		ANOSIM	
		Pseudo-*F*	*P*-value	R	*P*-value
Bacteria	Season	26.595	**<0.001**	0.634	**<0.001**
	Region	2.456	**0.045**	−0.001	0.47
	Season × Region	12.123	**<0.001**	—	—
Archaea	Season	2.393	0.103	0.030	0.105
	Region	3.574	**0.044**	−0.059	0.910
	Season × Region	2.464	**0.046**	—	—
Fungi	Season	15.045	**<0.001**	0.891	**<0.001**
	Region	2.505	**0.010**	0.110	**0.012**
	Season × Region	7.542	**<0.001**	—	—

Tests on bacteria and archaea were based on weighted UniFrac distances whereas Bray-Curtis distances were used for fungi. *P*-values in bold are significant at α = 0.05. ANOSIM does not calculate interaction effects.

**Table 2:** Effects of major explanatory variables on microbial communities.

Kingdom	Variable	Variance explained	Pseudo-*F*	VIF	*P*-value
Bacteria	Temp	0.309	23.713	1.368	0.001***
	ShnArc	0.055	4.237	1.381	0.002**
	TC	0.053	4.088	1.115	0.005**
	ObsFun	0.035	2.697	1.228	0.034*
	Final model	0.453	8.684	—	0.001***
Archaea	ObsFun	0.129	7.063	1.208	0.001***
	ShnBac	0.069	3.792	1.208	0.012*
	Final model	0.198	5.428	—	0.001***
Fungi	Temp	0.226	14.116	3.751	0.001***
	ObsBac	0.052	3.223	3.170	0.001**
	ShnArc	0.036	2.233	1.488	0.021*
	Final model	0.313	6.528	—	0.001***

Abbreviations: VIF, variance inflation factor; Temp, temperature; Shn, Shannon diversity; Obs, number of observed OTUs; Bac, bacteria; Arc, archaea; Fun, fungi. Tests on bacteria and archaea were based on weighted UniFrac distances whereas Bray-Curtis distances were used for fungi. Final model includes all listed explanatory variables. Significance was tested by ANOVA: *P < 0.05, **P < 0.01, ***P < 0.001.

For archaea, no distinct grouping of samples could be observed in the PCoA plot (Fig. 2b). Statistical tests also revealed no effect of season and only weak effects of sampling region (PERMANOVA, Pseudo-*F* = 3.574; ANOSIM, R = −0.059) and their interaction term (PERMANOVA, Pseudo-*F* = 2.464) on the archaeal community (Table 1). Constrained db-RDA resulted in a model that could barely explain 19.8% of the total variation of the archaeal community (Table 2, Fig. 3b). Fungal richness and bacterial diversity, explaining respectively 12.9% and 6.9% of the variation, were the major explanatory variables revealed. However, none of the abiotic factors examined here had significant effects on shaping the archaeal community (Supplementary Table S3).

Fungal samples were grouped by season and the season × region interaction term (Fig. 2c, Table 1). Significant but weak effect of sampling region (PERMANOVA, Pseudo-*F* = 2.505; ANOSIM, R = 0.110) on fungal community was also observed. Constrained db-RDA resulted in a model that could explain 31.3% of the total variation of the fungal community (Table 2, Fig. 3c). Similar to the case of bacteria, temperature was found to be the most important factor that shapes the fungal community, explaining 22.6% of the variation. Other important factors included bacterial richness (5.2%) and archaeal diversity (3.6%). Results of db-RDA indicated that seasonal difference of the fungal community in both mangrove and mudflat sediments could be explained by temperature, whereas that in mangrove sediments could additionally be explained by bacterial richness (Fig. 3c). TC and pH, explaining respectively 3.4% and 2.6% of the variation, were only significant in the model with all explanatory variables (Supplementary Table S3).

### Co-occurrence network analysis

#### Taxonomic and modular structures of networks

**Figure 4:**
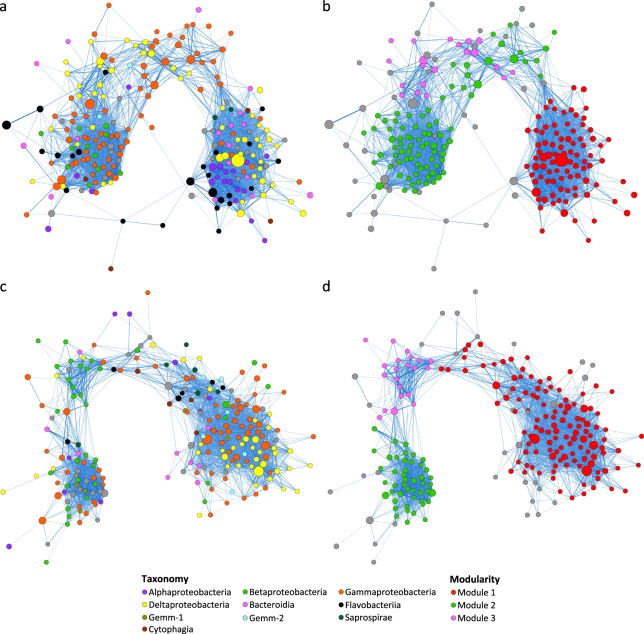
Co-occurrence networks built from major bacterial OTUs in sediments collected from the (**a,b**) dry and (**c,d**) wet seasons. Each node in the network represents an OTU whereas each edge represents a positive correlation. Nodes are coloured according to (**a,c**) class-level taxonomy or (**b,d**) modules predicted using the MCL algorithm. The size of nodes is proportional to the relative abundance of OTUs whereas the width of edges is proportional to the magnitude of correlation. Only edges with correlation values > 0.5 are shown here.

Bacterial co-occurrence network for the dry season comprised 229 core OTUs mainly from the classes Gammaproteobacteria, Deltaproteobacteria and Flavobacteriia (Fig. 4a). By contrast, bacterial co-occurrence network for the wet season comprised 221 core OTUs mainly from the classes Gammaproteobacteria, Deltaproteobacteria and Betaproteobacteria (Fig. 4c). Markov cluster (MCL)-based modular analysis revealed the presence of three distinct modules in each network (Fig. 4b,d).

**Figure 5:**
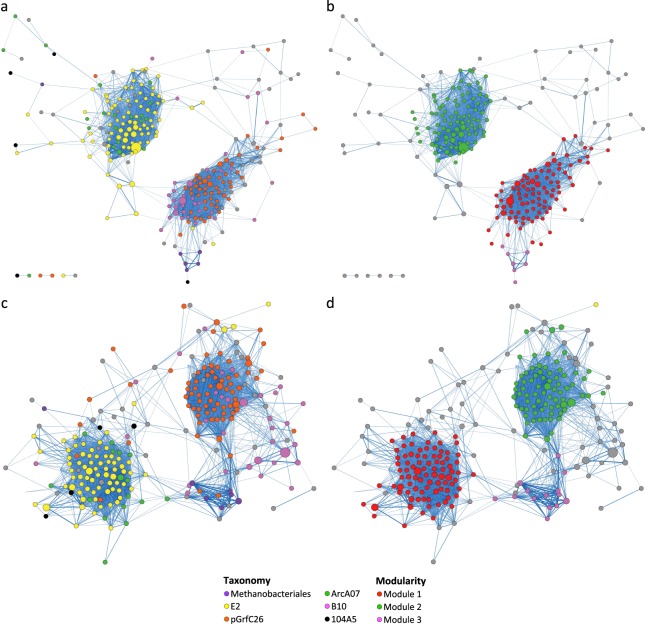
Co-occurrence networks built from major archaeal OTUs in sediments collected from the (**a,b**) dry and (**c,d**) wet seasons. Nodes are coloured according to (**a,c**) order-level taxonomy or (**b,d**) modules predicted using the MCL algorithm. Other details as in Fig. 4.

Archaeal co-occurrence network for the dry season comprised 249 core OTUs mainly from order-level groups E2, pGrfC26, B10, and ArcA07 (Fig. 5a). Similarly, archaeal co-occurrence network for the wet season comprised 252 core OTUs mainly from pGrfC26, E2, B10, and ArcA07 (Fig. 5c). Modular analysis revealed the presence of three modules that corresponded between the two networks (Fig. 5b,d).

**Figure 6:**
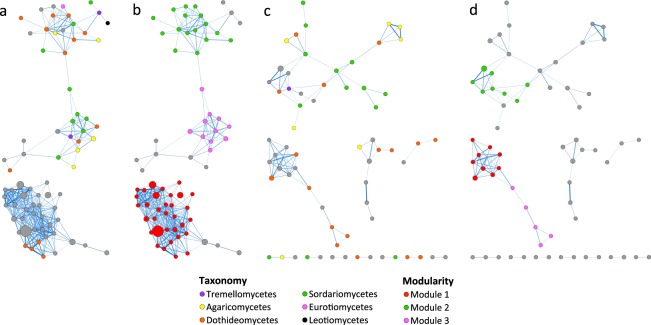
Co-occurrence networks built from major fungal OTUs in sediments collected from the (**a,b**) dry and (**c,d**) wet seasons. Nodes are coloured according to (**a,c**) class-level taxonomy or (**b,d**) modules predicted using the MCL algorithm. Other details as in Fig. 4.

Fungal co-occurrence network for the dry season (72 OTUs) mainly comprised unassigned OTUs from the phylum Ascomycota and from the classes Dothideomycetes and Sordariomycetes (Fig. 6a). Modular analysis revealed the presence of three distinct modules (Fig. 6b). Similarly, fungal co-occurrence network for the wet season (66 OTUs) mainly comprised OTUs that could not be assigned down to the class level and from the classes Dothideomycetes and Sordariomycetes (Fig. 6c). The top three modules resulted from the modular analysis were only small modules that contained 10 or fewer OTUs (Fig. 6d), in concordance with a non-modular organization of the network as suggested by the low value of clustering coefficient^15^ (Supplementary Table S4).

#### Correlations among taxa

Most highly positively correlated taxon pairs identified in the bacterial co-occurrence networks were members from the same family (Supplementary Tables S5a,b). Exceptions included, for instance, those between the families Piscirickettsiaceae and Sinobacteraceae. By contrast, most highly negatively correlated bacterial taxon pairs were members from different classes or even phyla (Supplementary Table S5a). Examples included those between the families Desulfuromonadaceae (class Deltaproteobacteria) and Sinobacteraceae (class Gammaproteobacteria), and between the families Flavobacteriaceae (phylum Bacteroidetes) and Ignavibacteriaceae (phylum Chlorobi).

Archaeal co-occurrence networks for both seasons revealed similar correlation relationships (Supplementary Tables S5c,d). There were strong positive correlations (magnitude > 0.5) between members within family-level groups Deep-Sea Hydrothermal Vent Euryarchaeotic Group I (DHVEG-1), WSA2, Methanomassiliicoccaceae, and 20c-4, as well as within the order-level pGrfC26 group. Strong positive correlation was also observed between pGrfC26 and B10 of the Bathyarchaeota phylum. By contrast, strong negative correlations were observed between pGrfC26 and members from DHVEG-1, Methanomassiliicoccaceae, CCA47, and Marine group III (class Thermoplasmata). Group B10 of Bathyarchaeota and DHVEG-1 of Euryarchaeota were also highly negatively correlated.

For fungi, strong positive correlations were observed between members within the order Pleosporales and the genus *Psathyrella* (Supplementary Tables S5e,f). By contrast, strong negative correlations were observed between the genus *Stagonosporopsis* and other unclassified members of the order Pleosporales, and between the genus *Fusarium* and the order Pleosporales.

#### Putative keystone taxa

In the bacterial network for the dry season, top putative keystone OTUs included members from the genera *Gallionella* and *Arcobacter*, and from the families Piscirickettsiaceae, Flavobacteriaceae, Sinobacteraceae, Desulfuromonadaceae, and Oceanospirillaceae (Supplementary Table S6a). By contrast, putative keystone OTUs in the wet season network included two OTUs belonging to the genus *Nitrospira* and an OTU belonging to the family Marinicellaceae. A putative keystone OTU that could only be assigned down to the Proteobacteria phylum (Bac.NR.OTU1333) was present in both networks.

For archaea, top putative keystone OTUs in the dry season network comprised members from DHVEG-1, Methanomassiliicoccaceae and pGrfC26 (Supplementary Table S6b). Similarly, putative keystone OTUs in the wet season network included members from DHVEG-1 and pGrfC26. Indeed, four of the pGrfC26 OTUs (Arc.NR.OTU73, Arc.NR.OTU208, Arc.NR.OTU218, and Arc.NR.OTU388) were shared in both networks.

Majority of the putative keystone OTUs in the fungal networks could not be identified beyond the phylum level (Supplementary Table S6c). Nevertheless, members from the genus *Massarina* and the family Psathyrellaceae were putative keystone OTUs in the fungal network for the dry season whereas putative keystone OTUs in the wet season network included members from the orders Pleosporales and Tremellales.

## Discussion

The archaeal community was mainly composed of the phyla Bathyarchaeota and Euryarchaeota here. Bathyarchaeota, formerly named as the Miscellaneous Crenarchaeotic Group (MCG)^16^, represents a diverse group of deep-branching archaea comprising at least 21 subgroups^17,18^. In particular, subgroups 6 and 8 members recovered here are frequently found in tidal flat, salt marsh and estuary sediments^17^. Metagenomics studies have shown that subgroup 6 members of Bathyarchaeota are organo-heterotrophic and autotrophic acetogens^19^ and that subgroup 8 members have the potential for methylotrophic methanogenesis using a wide range of methylated compounds^20^. Euryarchaeota was dominated by DHVEG-1 here, members of which have been discovered in salt marsh subsurface sediments and continental shelf anoxic sediments^21^. Recent studies have shown that DHVEG-1 members are capable to perform acetogenesis^22^. These results show that numerous uncultured archaeal groups are present in anoxic coastal wetland surface sediments and play crucial roles in methane and carbon cycling. It is noteworthy that the phylum Thaumarchaeota, which includes chemolithoautotrophic ammonia oxidizers that have a ubiquitous distribution^23^, represented an average of only 1% in our samples. A low relative abundance of Thaumarchaeota (~0.5%) has also been reported in mangrove sediments in Brazil^5^. The low contribution of Thaumarchaeota detected here may be related to the low salinity (<19 PSU) of the sampling sites examined in this study as the distribution of Thaumarchaeota has been shown to vary with salinity, with higher abundance at sites with higher salinity and lower abundance at sites with lower salinity^6^.

The fungal community was overwhelmed by members from the Ascomycota phylum here, in agreement with studies in other mangrove and salt marsh sediments^8,24^. Dominant fungal orders recovered here included Pleosporales, Hypocreales, Agaricales, Capnodiales, and Sordariales, all of which comprise members representing saprobes and/or plant pathogens and contain known marine members^25^. The retrieval of an abundant amount of potentially marine-derived saprotrophic fungi here provides support to the previous claim that marine fungi play crucial roles in the recycling of organic matter in the organic-rich coastal wetland ecosystem^26^. Here, an average of 23% of the ITS reads cannot be assigned to a known fungal phylum. Indeed, over 60% of ITS reads recovered from anoxic, sulphidic mangrove sediments in France can only match to uncultured fungal members too^24^. In another study, about 12% of fungal 28S ribosomal RNA (rRNA) reads recovered from salt marsh sediments were also unidentifiable to phylum or beyond^8^. As coastal wetlands are important transition zones between terrestrial and marine ecosystems, these cryptic fungi may represent novel early diverging lineages that are important for us to understand the evolution of fungi^7^. Extended sampling in other coastal wetlands and sophisticated phylogenetic analysis are required to better characterize these cryptic fungal lineages in the coastal wetland ecosystems.

Bacterial communities in mangrove and mudflat sediments examined here differed significantly between seasons, in agreement with studies on other wetlands^27,28^. Fungal communities also showed seasonal difference in composition here, although fungal richness and diversity remained relatively stable between seasons. Among all abiotic factors tested, temperature was found to be a major driver that could explain the seasonal patterns of bacterial and fungal communities here. Temperature affects microbial growth and activity by changing the rate of enzyme activity and the efficiency of membrane transport^29^, as well as altering the temperature-dependent growth rate of the microbial communities^30^. It was also reported as an important driver of bacterial community in temperate mudflat sediments^27^ and fungal community in freshwater sediments^31^. Significant variations of bacterial and fungal communities between mudflat and mangrove sediments were only detected in the dry season. Spatial variations of bacterial and fungal communities between vegetated and non-vegetated sites were also reported in temperate mudflat^27^ and marsh sediments^32^, respectively. Differential patterns in microbial communities observed between seasons here may be related to difference in seasonal rainfall intensity. It has been shown that rainfall events during low tide can mobilise and transfer large volumes of marsh sediments by sheetflow erosion^33^. Thus, the larger amount of precipitation in the wet season here may have attenuated potential spatial difference between surface sediments from the two sampling regions.

Unlike bacteria and fungi, archaeal communities remained relatively stable between sampling regions and seasons here. These findings are in contrast with our initial hypothesis, as both vegetation and season were expected to exert significant effects on the archaeal community turnover, as reported in other types of wetland^12^. It has been suggested that low-growing soil microorganisms such as Bathyarchaeota^34^, which was abundant here, and methanogens^35^ are less affected by dry/wet cycles^36^. Alternatively, the archaeal members may have responded through physiological adaptations that are not detectable by the examination of the 16S rRNA gene alone^2^. Neither temperature nor any other abiotic factors tested here was found to be a major driver of the archaeal community structure. A previous meta-analysis has also shown that temperature is not a driver of global ecological patterns in uncultured archaea^37^. Archaeal communities in forest soils^38^ and grassland soils^14^ are also largely unresponsive to abiotic factors. Archaea often represent only a small proportion of total microbial communities in soils and sediments^6,17^. According to the population-genetic theory, when the population size is small, selection is usually ineffective^39^, relatively stable distribution patterns may then result. However, the possibility remains that the archaeal communities are controlled by factors that were not examined in these studies. Seasonally stable archaeal communities were also reported in methanogen-dominated boreal fen^40^ and freshwater wetland soils^41^. Therefore, it appears that the overall effects of season and vegetation on archaeal communities in wetland sediments differ among wetland types, and possibly depend on the phylogenetic and functional groups of archaea present, as illustrated in the case of fungi^42^. This disparity also illustrates the need to characterize a wider range of wetlands to acquire a comprehensive understanding of microbial distribution.

Based on the db-RDA results from all three kingdoms, a conceptual model of abiotic and biotic factors shaping the microbial communities in subtropical wetland sediments examined here was built (Fig. 3d). Our model highlights the importance of interkingdom biotic factors, along with abiotic factors such as temperature and TC, as drivers of microbial communities in the sediments. Abiotic drivers as principal driving forces of global ecological patterns of bacteria, fungi and uncultured archaea have been reported^37,42,43^. By contrast, biotic factors as drivers of microbial communities are often overlooked. A recent study has shown that archaeal abundance influenced bacterial community whereas bacterial diversity and archaeal richness influenced fungal community in grassland soils^14^. Other studies have shown that fungi were the major driver of bacterial assemblage^44^, and can alter archaeal community structure in soil^45^. These results show that biotic factors should be taken into consideration, along with abiotic factors, in future studies on elucidation of factors shaping microbial communities. However, it is noteworthy that data of some other potentially important variables, such as salinity, sediment granularity and inorganic nutrient contents, were not collected in this study. Previous studies have shown that salinity could affect the bacterial community structure in temperate mudflat sediments^27^, and is, along with sulphate content, an important driver of bacterial assemblages in salt marsh sediments^4^. Sand and clay were also reported to drive fungal community structures in salt marsh sediments^7^. Therefore, further studies should examine these factors, along with those examined here, to obtain a more comprehensive picture of the factors that drive the community structures of bacteria, archaea and fungi in subtropical mangrove wetland sediments.

Co-occurrence networks are a powerful tool for generating hypotheses about interactions that can then be tested in targeted experiments^10,46^. For all three kingdoms, most of the strong positive correlations revealed here were between members from the same families or orders. By contrast, most of the strong negative correlations were between members from different classes or even phyla. This agrees with previous observation that phylogenetically related microorganisms tend to co-occur^47^, possibly as a result of niche overlap^48^. Negative correlation patterns may simply result from a difference in niche preference. This may be illustrated by the strong negative correlations reported here between the bacterial families Desulfuromonadaceae and Sinobacteraceae, Desulfuromonadaceae and Piscirickettsiaceae, as well as Syntrophobacteraceae and Sinobacteraceae. In all these cases, the former groups comprise strictly anaerobic members whereas the latter groups are composed of aerobic ones. Competition of common resources is another possible reason that could lead to negative correlations. For instance, the strong negative correlations observed between members of pGrfC26, which have the ability to hydrolyse extracellular plant-derived carbohydrates and degrade detrital proteins^19^, and DHVEG-1, which are centred on the degradation of detrital proteins^22,49^, may indicate a competition of protein substrates.

In a concept initially developed for macroorganisms, keystone species are those that exert disproportionate large influences on the community relative to their abundance^50^. The discovery of microbial keystone species severely lags behind their macro-counterparts. Recently identified microbial keystone species included, for instance, the sulphate-reducing *Desulfosporosinus* in peatland soil^51^ and the amylolytic *Ruminococcus bromii* in the human colon^52^. Here, bacterial members from genera *Gallionella*, *Arcobacter* and *Nitrospira*, archaeal members from DHVEG-1 and pGrfC26, and fungal members from the genus *Massarina* were identified as putative keystone OTUs in the co-occurrence networks. *Gallionella ferruginea*, the sole known species of the genus, is a well-known neutrophilic iron-oxidizing bacterium^53^. It is suggested that iron-oxidizing bacteria may compete with oxidizers of ammonium, methane, and sulphide for limited O2 at the oxic/anoxic interface of wetland sediments, therefore influencing other wetland biogeochemical cycles^54^. *Nitrospira* is a ubiquitous and diverse genus of nitrite-oxidizing bacteria. Recent metagenomic studies have recovered several *Nitrospira* strains capable of performing complete nitrification on their own^55^, and point to these complete ammonia oxidizers (‘comammox’) as previously overlooked key components of nitrogen-cycling microbial communities. Both DHVEG-1 and pGrfC26 are potential acetogens^19,22^. The metabolic versatility of acetogens to convert a large variety of substrates to acetate links fermentative bacteria to methanogens, which can use acetate as a substrate, and makes acetogens an essential part of anaerobic food webs^56^. Indeed, the detection of pGrfC26 members as putative keystone OTUs here agrees with previous suggestion that members of the Bathyarchaeota subgroup, to which pGrfC26 members belong, have a keystone role in archaeal sediment communities^18^.

In summary, this study represents the first detailed and simultaneous assessment of the structure, dynamics and interactions of bacterial, archaeal and fungal communities in mangrove and mudflat sediments of subtropical coastal wetland. Such information is essential to better understand the microbial ecology of wetlands and to improve Earth-system models^1^, especially considering the continuous climate change-driven poleward expansion of subtropical mangrove^9^. Distinct spatiotemporal patterns observed among the three kingdoms imply different community assembly mechanisms. Redundancy analysis highlights the importance of often-overlooked biotic factors as drivers of microbial communities. Potential ecological interactions, in particular between uncultured archaeal groups, and putative keystone taxa revealed by network analysis have provided directions for more focused studies. Future validation studies, for instance with co-culture experiments^48^, will help disentangle the exact reasons behind the observed patterns.

## Methods

### Study site and sample collection

Sediments were collected from Mai Po wetland located on the northwestern part of subtropical Hong Kong (22°29′N, 114°01′E). Mai Po is the largest intertidal wetland in the Pearl River estuary of southern China and has been designated as a wetland of international importance under the Ramsar Convention since 1995. It is a natural shallow estuary that receives influence from Shenzhen River and inland San Pui River. The average water depth is about 2.9 m and the mean tidal range is 1.4 m. Tides in the wetland are mixed and mainly semi-diurnal. Mudflat and mangrove in the intertidal region represent two major habitats in the wetland. The seaward bare mudflat harbours abundant epifauna such as mudskippers and is the main feeding ground for migratory waterbirds, whereas the landward mangrove forest in Mai Po is dominated by *Kandelia obovata*.

Sediment samples were collected on 11 September 2015 and 7 March 2016, which represent the wet and dry season of subtropical Hong Kong, respectively. During August to September 2015, the average air temperature of the study area was 28.5 °C and total precipitation was 422.0 mm. By contrast, the average air temperature in February to March 2016 was 16.2 °C and total precipitation was 165.5 mm (Sheung Shui Weather Station, Hong Kong Observatory Online Weather Data). The average surface water temperature and salinity of the study area was 30.3 °C and 12 PSU, respectively, during August to September 2015 and 15.6 °C and 18.3 PSU, respectively, during February to March 2016 (DM1 station, Hong Kong Environmental Protection Department Marine Water Quality Data). The salinity of the mudflat region is generally lower than that of the mangrove region as a result of freshwater input from neighbouring rivers.

**Figure 7:**
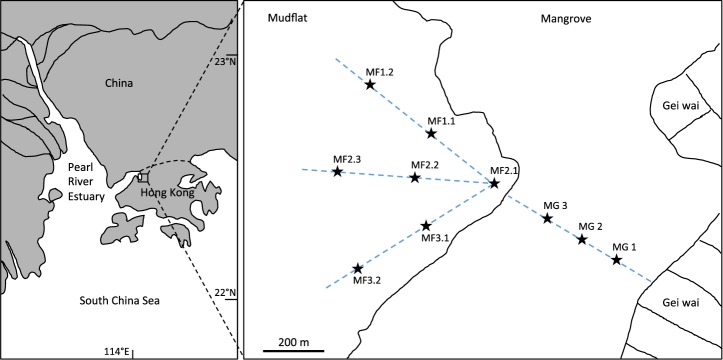
Map of sampling locations in Mai Po wetland. MF: mudflat, MG: mangrove.

Bulk surface (top 2 cm) sediment samples were collected in triplicate from three sites in the mangrove region (MG) and seven sites along three cross-shore transects in the larger mudflat region (MF) at low tide (<1.5 m) (Fig. 7). In total, 60 sediment samples were examined in this study. This sample set covers a gradient of environmental conditions and thereby provides a sufficient variability in taxon abundance for co-occurrence analysis^10^. Consecutive sampling sites were ~200 m apart from each other. In each sampling site, about 500 g of surface sediments were collected in triplicate within a 100 cm^2^ area and sieved through a 2-mm mesh to remove roots and large debris. Bulk mangrove sediment samples were collected at distance from the roots to avoid sampling of rhizosphere sediments. The collection instruments were sterilized with 70% ethanol between samplings. Sediment samples were put into sterile plastic bags, stored in ice, and returned to the laboratory within an hour. In the laboratory, 10 g of sediment was added to 10 ml DI water (1:1 v/v) and mixed to create a slurry, from which pH and redox potential (ORP) were measured with a HI 98121 pH/ORP/temperature combo tester (Hanna instruments, Woonsocket, RI, USA). Another portion of each sediment sample was stored at −80 °C until DNA extraction. Sediment subsamples (~2 g) for total carbon (TC), total nitrogen (TN) and total sulphur (TS) content analysis were oven dried at 105 °C overnight and then analysed using a vario MICRO cube elemental analyser (Elementar, Langenselbold, Hesse, Germany).

### DNA extraction, PCR and Ion Torrent sequencing

Genomic DNA was extracted from the sediments using the PowerSoil DNA Isolation Kit (MO BIO Laboratories, Carlsbad, CA, USA). The manufacturer’s protocol was slightly modified by using TissueLyser (QIAGEN, Germantown, MD, USA) for 1 min per side at 30 Hz to improve cell lysis and homogenization. PCR amplifications were performed using primers flanking hypervariable regions of the bacterial and archaeal 16S rRNA genes, as well as the nuclear ribosomal ITS region of fungi. For bacteria, the V1-V2 region of the 16S rRNA gene was amplified using primers 28F (5′-GAG TTT GAT CNT GGC TCA G-3′)^57^ and 338R (5′-GCT GCC TCC CGT AGG AGT-3′)^58^; for archaea, the V1-V2 region of the 16S rRNA gene was amplified using primers 21F (5′-TTC CGG TTG ATC CYG CCG GA-3′)^59^ and Pro341R (5′-CTG STG CVN CCC GTA GG-3′)^60^; and for fungi, the ITS1 region was amplified using primers ITS1F (5′-CTT GGT CAT TTA GAG GAA GTA A-3′)^61^ and ITS2 (5′-GCT GCG TTC TTC ATC GAT GC-3′)^62^. Phusion High-Fidelity DNA polymerase (New England Biolabs, Ipswich, MA, USA) was used for robust amplifications. Each 20 μl PCR reaction mix contained 1X Phusion HF Buffer, 0.5 μM primers, 200 μM dNTPs, 1 U of Phusion DNA Polymerase, and ~50 ng of DNA template. The thermocycling regime of the bacterial 16S rRNA gene consisted of an initial denaturation at 98 °C for 3 min, followed by 30 cycles of denaturation at 98 °C for 10 s, annealing at 61 °C for 30 s and extension at 72 °C for 30 s, and a final extension of 72 °C for 10 min. For archaea, an annealing temperature of 67 °C was used; and for fungi, an annealing temperature of 57 °C and 35 cycles were used. Triplicate PCR reactions were set up for each sample to minimize PCR bias. PCR products of each sample were then pooled, verified with electrophoresis on 1.5% agarose gels and purified using the QIAquick Gel Extraction Kit (QIAGEN, Germantown, MD, USA). The PCR products were further cleaned and size-selected (400 bp) using the Agencourt Ampure XP Kit (Beckman Coulter, Brea, CA, USA) following the Ion Torrent’s protocol. DNA concentration and quality of the purified PCR products were determined using an Agilent Bioanalyzer 2100 (Agilent Technologies, Santa Clara, CA, USA) and a Qubit 2.0 Fluorometer (Thermo Fisher Scientific, Waltham, MA, USA). For each kingdom, purified PCR products of sufficient quality and quantity were pooled at equimolar concentrations and sequenced together unidirectionally from the forward primer end on an Ion Torrent PGM system (318 chip v2) at the Core Facilities of the Chinese University of Hong Kong.

### Sequence analysis

Raw sequence reads were demultiplexed, filtered for quality and analysed using QIIME 1.9.1^63^ as previously described^64^. Chimeric sequences were identified and removed using USEARCH 6.1^65^ against the “Gold” reference dataset for prokaryotes and the UNITE dynamic ITS1 reference dataset (2016–01–01 release)^66^ for fungi. Reads from the same kingdom were clustered into operational taxonomic units (OTUs) at 97% similarity using uclust^65^ with the open-reference OTU picking approach. Representative OTUs were aligned to the Greengenes reference dataset (13_8 release) for prokaryotes^67^ and the UNITE dynamic reference dataset (2016–11–20 release) for fungi. Taxonomic assignment of the representative OTUs was performed using the uclust consensus taxonomy assigner^65^ based on a similarity threshold of 0.8 for prokaryotes and BLAST for fungi. Reads of chloroplast and non-fungal origin were removed from the prokaryote and fungal datasets, respectively. The archaeal taxonomy was manually curated with reference to the review by Spang *et al*.^68^.

### Statistical analysis

Samples with fewer than 20,000 reads were removed from subsequent analyses. Sequence datasets were rarefied to the smallest sample before diversity analyses. Alpha diversity estimates were calculated for each sample and compared among four sample groups defined by a combination of season (wet or dry) and region (MG or MF) using the non-parametric Monte Carlo method (999 permutations). Unconstrained principal coordinates analysis (PCoA) for prokaryotes and fungi were performed using weighted UniFrac and Bray–Curtis distances, respectively. Seasonal and spatial effects on the microbial communities were tested using permutational multivariate analysis of variance (PERMANOVA) and analysis of similarities (ANOSIM) (99,999 permutations). Statistical difference in relative abundance of taxa between sample groups was tested using Kruskal–Wallis tests with *P*-values corrected by the false discovery rate method of Benjamini and Hochberg^69^. All the above statistical tests were conducted using scripts in QIIME. Unconstrained non-metric multidimensional scaling (NMDS) was performed using the *metaMDS* function (k = 3) of the *vegan* package 2.4-1 in R version 3.3.2 (http://www.r-project.org/) to examine potential arch effects in PCoA plots. Constrained distance-based redundancy analysis (db-RDA) was also carried out using the *capscale* function of the *vegan* package to determine factors that could best explain variations in the microbial communities. The analysis was based on subsample sets for which complete data sets of explanatory and response variables were available. Explanatory variables tested were standardized using z-scoring and included pH, ORP, TC, TN, TS, and temperature (abiotic factors), and richness and Shannon diversity of bacteria, archaea and fungi (biotic factors). Significance of the models and the explanatory variables were tested by analysis of variance (ANOVA) with 999 permutations. To avoid overfitting, only statistically significant (*P* < 0.05) and non-collinear (variance inflation factor <4) variables were included in the final biplots.

### Network analysis

Co-occurrence networks were built for each of the three kingdoms in each season. To increase network sensitivity, OTUs found in <50% of samples or with an average relative abundance of <0.05% were discarded^46^. Correlations between OTUs were then calculated using SparCC^47^ based on 20 iterations and 500 bootstraps. Only statistically significant edges (p < 0.001) with correlation values > 0.5 or <−0.5 were kept^70^. Networks constructed were visualized with the organic layout and annotated using Cytoscape 3.4.0. Network topology parameters were calculated using the built-in NetworkAnalyzer plugin. Modules within networks were identified with the MCL algorithm^71^ using default settings in the clusterMaker plugin^72^. Putative keystone OTUs in the networks were identified by calculating a keystoneness score from the average of degree, 1−betweenness centrality and closeness centrality after Min-Max scaling^46^.

## Electronic supplementary material

Supplementary Material

Table S5

Table S6

## Data Availability

The sequencing datasets generated for this study can be found in the NCBI Sequence Read Archive under study number SRP113428.
