# Supplementary material for: Community Structure, Dynamics and Interactions of Bacteria, Archaea and Fungi in Subtropical Coastal Wetland Sediments
Source: Sci Rep. 2018 Sep 26;8:14397. doi: 10.1038/s41598-018-32529-5 (PMC6158284; doi:10.1038/s41598-018-32529-5)
Supplement: Supplementary file 1 — Supplementary Material [file 41598_2018_32529_MOESM1_ESM.pdf]

Community Structure, Dynamics and Interactions of Bacteria, Archaea and Fungi in Subtropical Coastal Wetland Sediments

Man Kit Cheung[1], Chong Kim Wong[1], Ka Hou Chu[1], and Hoi Shan Kwan[1*]

[1]School of Life Sciences, The Chinese University of Hong Kong, Hong Kong SAR, China

***Correspondence:**
Prof. Hoi Shan Kwan
hoishankwan@cuhk.edu.hk

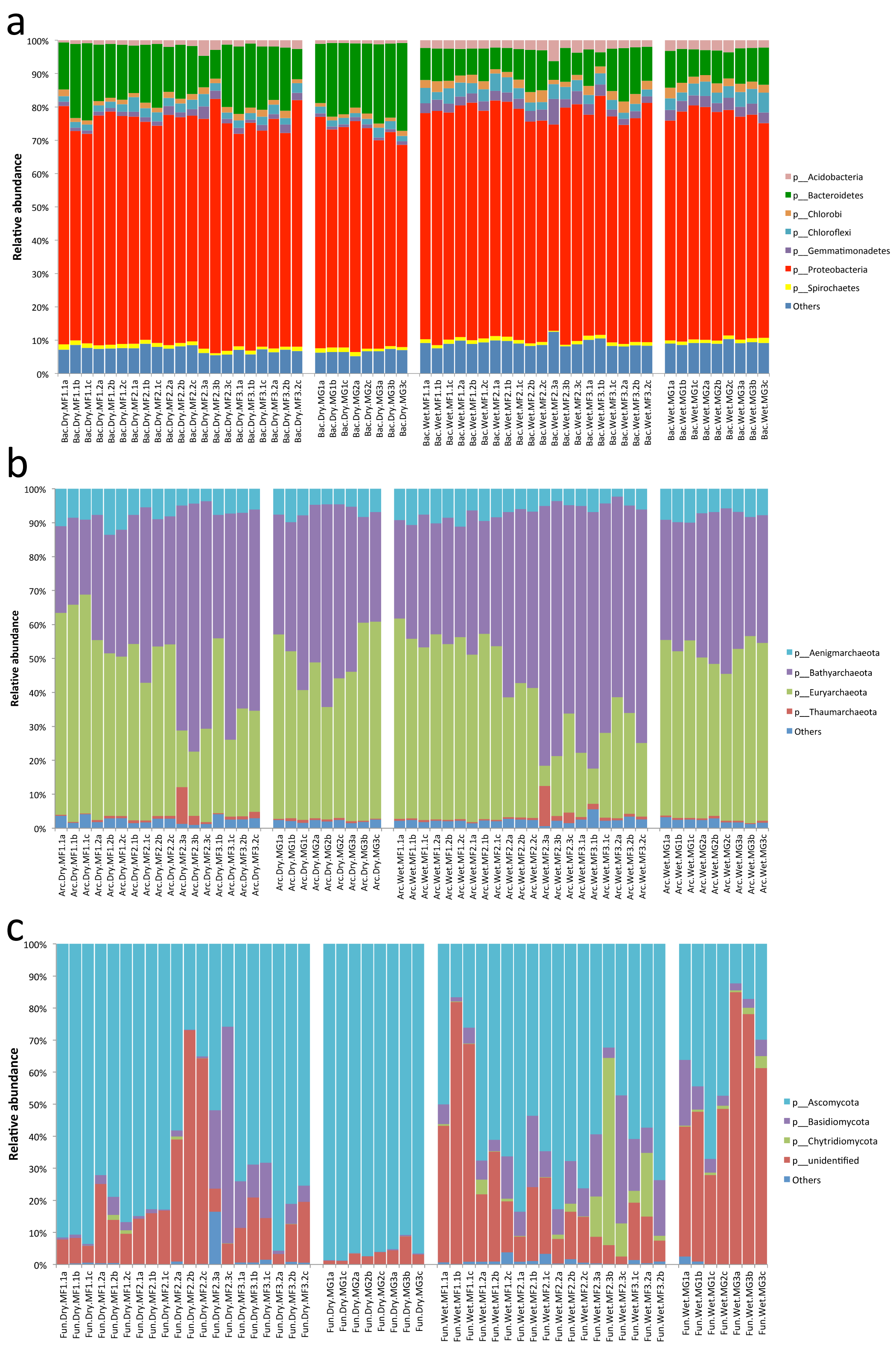

Figure S1. Relative abundance of dominant phyla of (a) bacteria, (b) archaea, and (c) fungi according to the number of sequence reads. Only groups with an average relative abundance ≥1% are shown here. Dry: dry season, Wet: wet season; MF: mudflat, MG: mangrove.

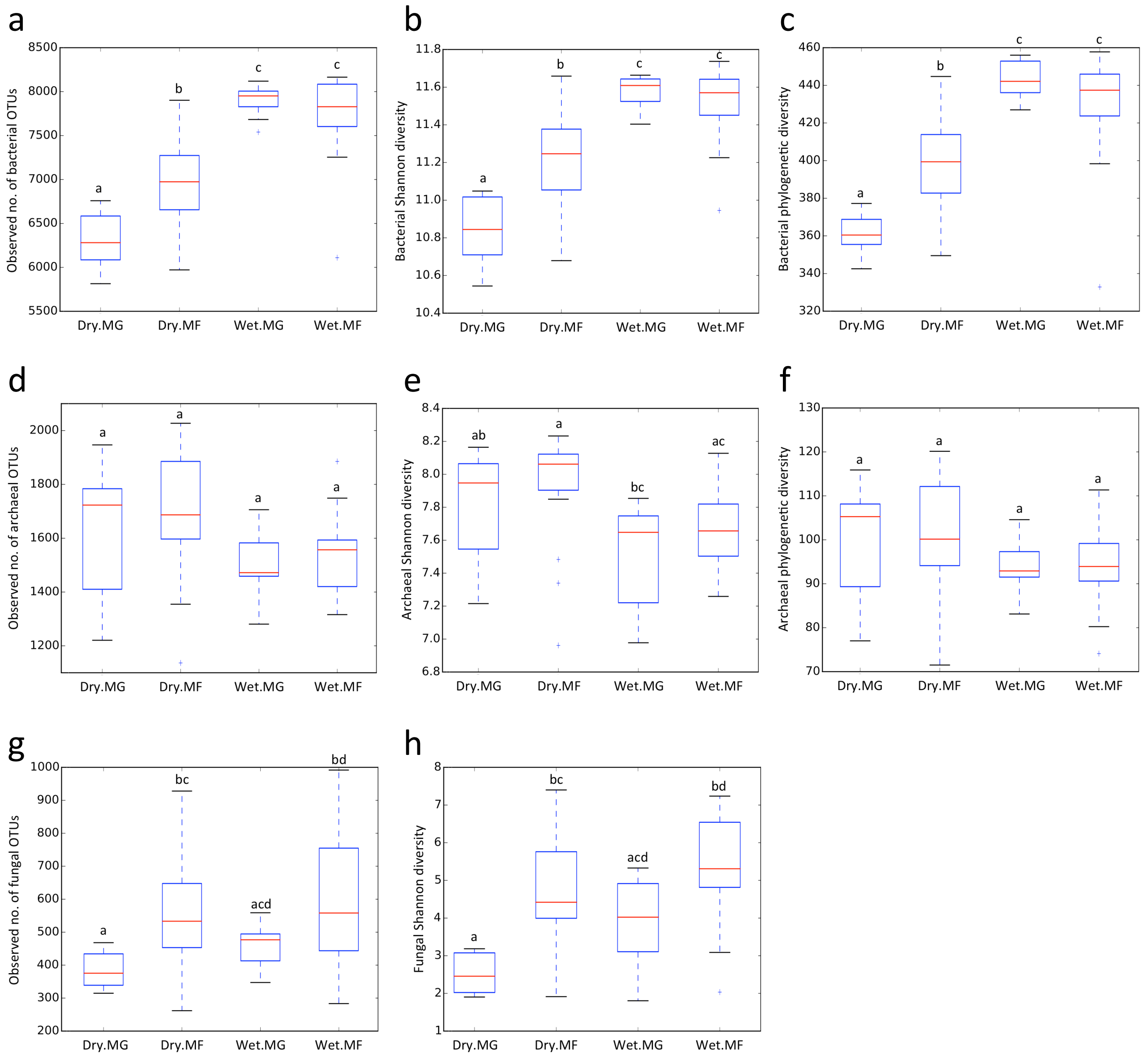

Figure S2. Alpha diversity estimates of (a-c) bacterial, (d-f) archaeal, and (g-h) fungal communities. The bacterial, archaeal and fungal datasets were rarefied to 20,310 reads, 20,300 reads, and 20,250 reads, respectively, before calculations. Different letters indicate statistically different means at $P<0.05$. MF: mudflat, MG: mangrove.

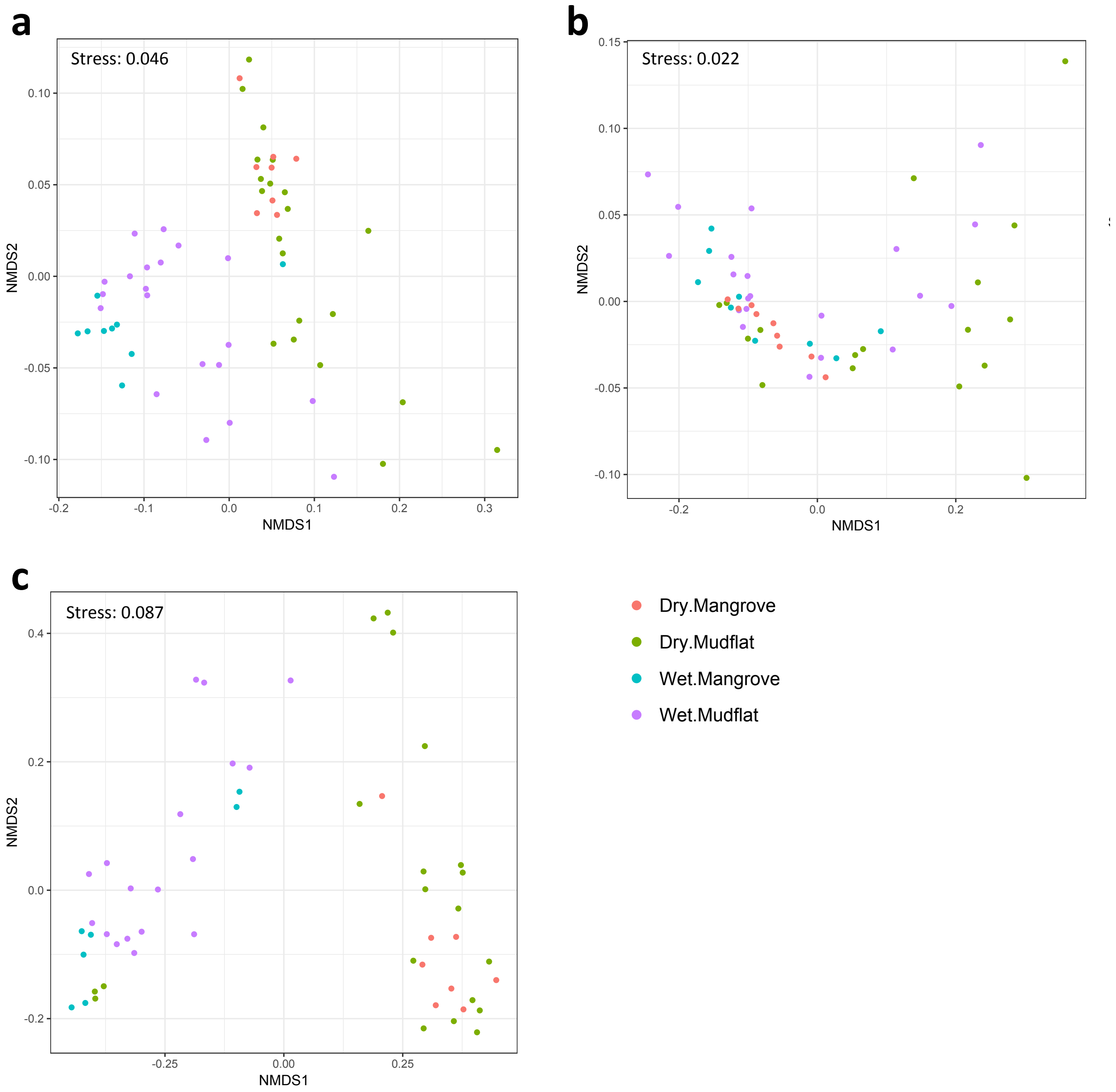

Figure S3. Non-metric multidimensional scaling (NMDS) plots of (a) bacterial, (b) archaeal and (c) fungal communities. Weighted UniFrac distances were used for bacteria and archaea whereas Bray-Curtis distances were used for fungi. Dry: dry season, Wet: wet season.

Table S1. Physico-chemical parameters of Mai Po wetland surface sediments

Season	*Region*	*n*	*pH*	*ORP (mV)*	*TC (%)*	*TN (%)*	*TS (%)*
Dry	Mudflat	21	7.48 (±0.15)[a]	-125.48 (±14.37)[a]	1.45 (±0.59)[a]	0.13 (±0.02)[a]	0.86 (±0.15)[a]
	Mangrove	9	6.61 (±0.40)[b]	65.78 (±67.68)[b]	3.05 (±1.33)[b]	0.19 (±0.08)[b]	0.65 (±0.18)[b]
Wet	Mudflat	21	7.48 (±0.15)[a]	-99.14 (±22.41)[c]	1.06 (±0.69)[a]	0.11 (±0.01)[c]	0.81 (±0.13)[a]
	Mangrove	9	6.74 (±0.36)[b]	22.89 (±99.42)[b]	2.29 (±0.92)[b]	0.22 (±0.06)[b]	0.59 (±0.09)[b]

Abbreviations: n, number of samples; ORP, redox potential; TC, total carbon; TN, total nitrogen; TS, total sulphur. Values are presented as mean (± standard deviation). Small letters indicate statistically significant differences ($P<0.05$) between individual means assessed by t-tests.

Table S2. Summary of alpha diversity statistics

Kingdom	*Season*	*Region*	*n*	*Obs*	*Shannon*	*PD*	*Good*
Bacteria	Dry	Mudflat	21	6,956 (±483)	11.20 (±0.25)	396 (±25)	0.77
		Mangrove	8	6,308 (±303)	10.84 (±0.17)	361 (±10)	0.79
	Wet	Mudflat	20	7,733 (±457)	11.51 (±0.19)	429 (±28)	0.73
		Mangrove	9	7,886 (±170)	11.58 (±0.08)	443 (±9)	0.73
Archaea	Dry	Mudflat	17	1,713 (±237)	7.93 (±0.34)	102 (±13)	0.96
		Mangrove	9	1,626 (±240)	7.80 (±0.35)	99 (±13)	0.96
	Wet	Mudflat	21	1,535 (±143)	7.66 (±0.22)	94 (±8)	0.96
		Mangrove	9	1,507 (±113)	7.51 (±0.32)	94 (±6)	0.96
Fungi	Dry	Mudflat	20	556 (±159)	4.63 (±1.38)	—	0.99
		Mangrove	8	385 (±55)	2.52 (±0.50)	—	0.99
	Wet	Mudflat	18	603 (±202)	5.38 (±1.40)	—	0.99
		Mangrove	7	457 (±67)	3.89 (±1.22)	—	0.99

Abbreviations: n, number of samples; Obs, number of observed OTUs; Shannon, Shannon diversity index; PD, phylogenetic diversity; Good, Good's coverage. Datasets of bacteria, archaea and fungi were normalized to 20,310 reads, 20,300 reads and 20,250 reads per sample, respectively. Alpha diversity measures are presented as mean (± standard deviation). PD values were not calculated for fungi because ITS-based phylogenetic tree of fungi is generally not considered informative.

Table S3. Effects of all explanatory variables on microbial communities

Kingdom	*Variable*	*Variance explained*	*pseudo-F*	*P-value*
Bacteria	pH	0.019	1.653	0.147^{NS}
	ORP	0.016	1.349	0.224^{NS}
	TC	0.048	4.122	0.006**
	TN	0.024	2.065	0.086^{NS}
	TS	0.021	1.807	0.095^{NS}
	Temp	0.295	25.210	0.001***
	ObsArc	0.023	1.990	0.105^{NS}
	ShnArc	0.069	5.889	0.001***
	ObsFun	0.028	2.359	0.051^{NS}
	ShnFun	0.035	3.015	0.020*
	Final model	0.579	4.946	0.011***
Archaea	pH	0.017	1.147	0.270^{NS}
	ORP	0.010	0.679	0.550^{NS}
	TC	0.023	1.540	0.181^{NS}
	TN	0.024	1.572	0.148^{NS}
	TS	0.011	0.721	0.445^{NS}
	Temp	0.024	1.606	0.164^{NS}
	ObsBac	0.030	1.963	0.107^{NS}
	ShnBac	0.184	12.252	0.001***
	ObsFun	0.104	6.941	0.002**
	ShnFun	0.031	2.054	0.105^{NS}
	Final model	0.458	3.047	0.001***
Fungi	pH	0.026	1.731	0.045*
	ORP	0.025	1.670	0.070^{NS}
	TC	0.034	2.244	0.015*
	TN	0.027	1.748	0.057^{NS}
	TS	0.017	1.134	0.272^{NS}
	Temp	0.199	13.155	0.001***
	ObsBac	0.043	2.853	0.002**
	ShnBac	0.032	2.110	0.023*
	ObsArc	0.018	1.204	0.213^{NS}
	ShnArc	0.032	2.121	0.024*
	Final model	0.454	2.997	0.001***

Abbreviations: ORP, redox potential; TC: total nitrogen; TN: total nitrogen; TS: total sulphur; Temp, temperature; Obs: number of observed OTUs; Shn, Shannon diversity; Bac: bacteria; Arc: archaea; Fun: fungi. Tests on bacteria and archaea were based on weighted UniFrac distances whereas Bray-Curtis distances were used for fungi. Final model includes all listed explanatory variables. Significance was tested by ANOVA: *P<0.05, **P<0.01, ***P<0.001, ^{NS}nonsignificant.

Table S4. Network parameters of co-occurrence networks

	Bacteria		*Archaea*		*Fungi*	
	Dry	*Wet*	*Dry*	*Wet*	*Dry*	*Wet*
Number of nodes	229	221	249	252	72	66
Number of edges	3,605	3,012	4,126	5,005	361	101
Avg. number of neighbors	31.485	27.258	33.141	39.722	10.028	3.061
Network density	0.138	0.124	0.134	0.158	0.141	0.047
Connected components	1	1	4	1	2	11
Network diameter	8	10	11	8	8	6
Network radius	5	5	1	5	2	1
Clustering coefficient	0.668	0.650	0.617	0.716	0.628	0.353
Characterstic path length	3.220	3.521	3.433	3.232	2.636	2.865
Network centralization	0.188	0.233	0.166	0.162	0.246	0.094
Network heterogeneity	0.669	0.686	0.732	0.687	0.694	0.782
